# Seborrheic Dermatitis-Like Eruption Induced by Isotretinoin: A Case Report

**DOI:** 10.7759/cureus.38665

**Published:** 2023-05-07

**Authors:** Aymen Alharbi, Fawaz H Aljehani, Asmaa Siddique, Ashwaq K Alosaimi, Sultan Assiri

**Affiliations:** 1 Dermatology and Hair Disorders, King Abdulaziz Hospital, Makkah, SAU; 2 Dermatology, King Abdulaziz Hospital, Makkah, SAU; 3 Dermatology, King Abdulaziz Hospital, Makkah , SAU; 4 Dermatology, Al-Noor Speciallist Hospital, Makkah, SAU

**Keywords:** infantile eczema, adverse effects, isotretinoin, seborrheic dermatitis, acne

## Abstract

Isotretinoin has received widespread medical attention for the management of moderate to severe acne vulgaris. It has been associated with various dermatological side effects, particularly dryness and cheilitis. To our knowledge, only one study has provided evidence of isotretinoin-induced seborrheic dermatitis-like eruptions. In addition, other adverse effects of isotretinoin have been documented in the literature, such as angioedema and urticaria. Here, we present the case of an 18-year-old female with severely scarred acne vulgaris who developed a seborrheic dermatitis-like eruption shortly after starting isotretinoin. Two months after stopping the causative drug and adhering to the topical treatment, the patient showed full resolution.​​*`*This case led to the conclusion that using isotretinoin may have unanticipated serious side effects. It is crucial to identify this complication to prevent misdiagnosis and to appropriately and promptly treat the patient’s condition.

## Introduction

The association between isotretinoin and different dermatological side effects affecting the face has been documented in the literature. These side effects include angioedema [1-2], urticaria [2], perioral abscess [3], and lip abscess [4]. To our knowledge, seborrheic dermatitis-like eruptions have been narrowly described in the literature. One study done by Barzilai et al. in 2012 described five patients who presented with yellow, greasy scales on the cheeks that resembled seborrheic dermatitis during or after the course of isotretinoin for acne [5].

Since using isotretinoin could lead to unexpected major consequences, it is essential to recognize the very uncommon complications in order to avoid a delayed diagnosis and to commence the appropriate therapy promptly. Herein, we present the case of an 18-year-old female patient who experienced a seborrheic dermatitis-like eruption on her cheeks shortly after receiving isotretinoin for the treatment of her severe acne.

## Case presentation

**Figure 1:**
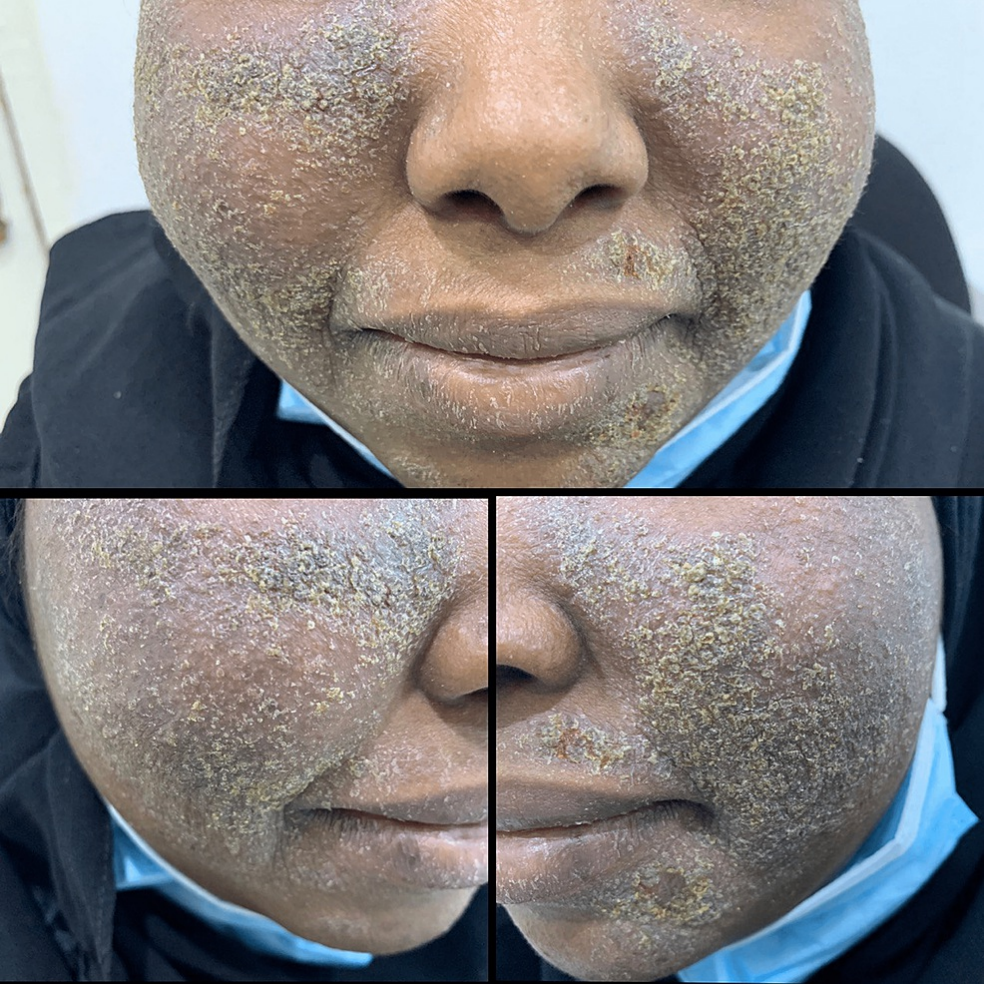
The patient at the time of presentation. Adherent, greasy, yellow, scaly plaques disseminated over both cheeks and the chin.

Ms. A, an 18-year-old female with infantile eczema in her medical history, was referred to our dermatology clinic for severe acne vulgaris grade lll since she was a 12 years old. She had no history of contact or food allergies. Due to the failure of topical treatments, a decision was made to start her on oral isotretinoin 20 mg once daily. Two months later, the dose of isotretinoin was increased to 40 mg once daily. A few days later, the patient presented to us with itchy, painful, adherent yellow scaly plaques with mild pus and bloody discharge, which started gradually in the periorbital area and forehead, expanding to reach the cheeks and perioral area predominantly (Figure 1). No other sites in the body were involved. She had no fever or any other systemic symptoms.

**Figure 2:**
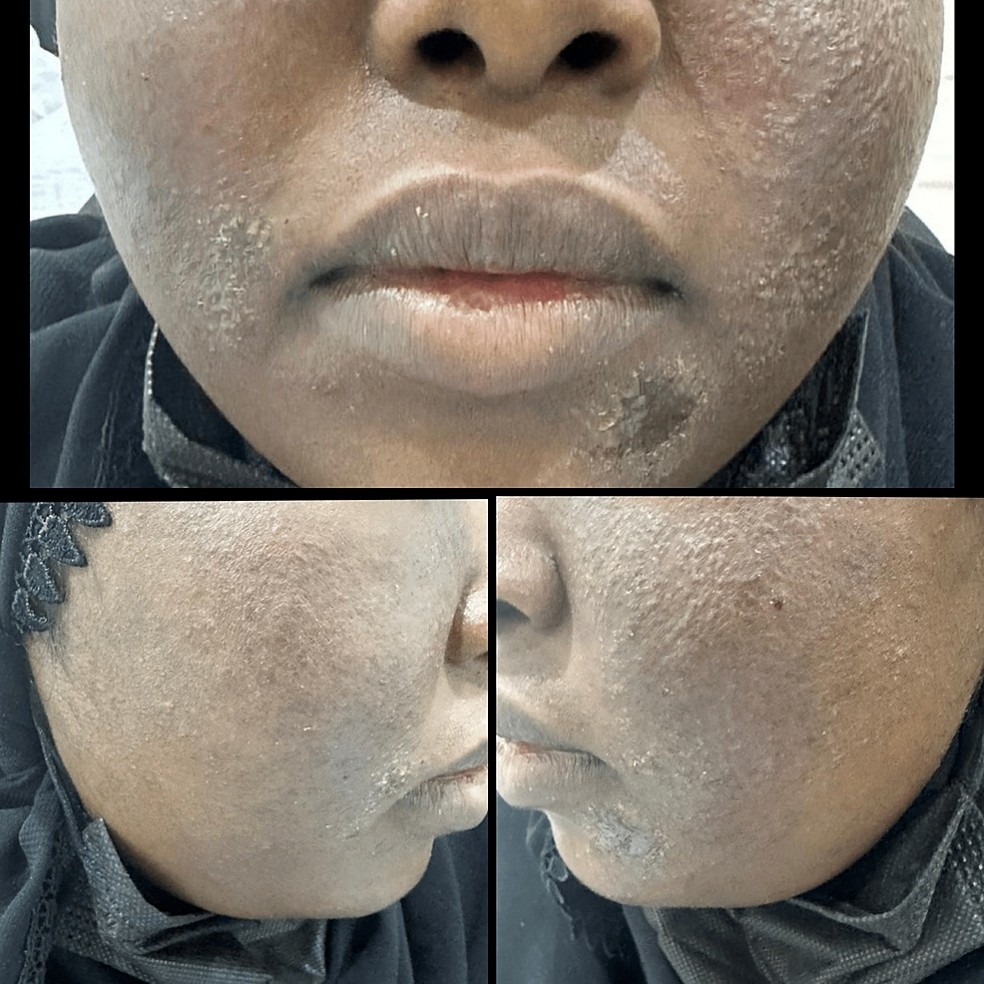
The patient two weeks after the start of treatment. Appreciable improvement can be seen in the scaly lesions, mainly over the cheeks.

**Figure 3:**
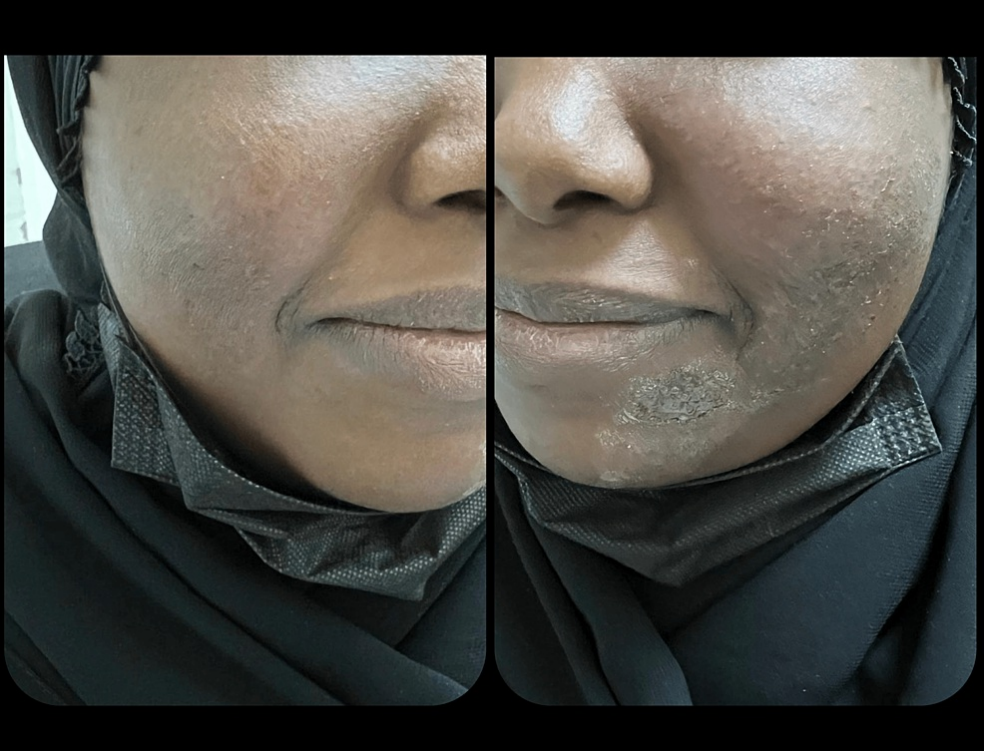
Six weeks after the start of the treatment. Almost complete resolution on the cheeks and to a lesser extent the chin.

As a consequence, isotretinoin was discontinued immediately. Given the impression that an infection might be present, a swab from the white discharge was taken. We started our patient immediately on a wide spectrum oral antibiotic, fusidic acid 2% cream, hydrocortisone cream 1% w/w, emollients, and potassium permanganate (KMnO_4_). Two weeks later, the symptoms largely improved. Swab results showed no evidence of a bacterial or fungal infection. Lesions over the forehead and periorbital area were fully resolved. However, lesions on the cheeks, chin, and perioral area were still slightly crusted adherent plaques (Figure 2). Four weeks later, the patient showed much improvement compared to the last visit (Figure 3). No hospital admission or intravenous medications were required.

**Figure 4:**
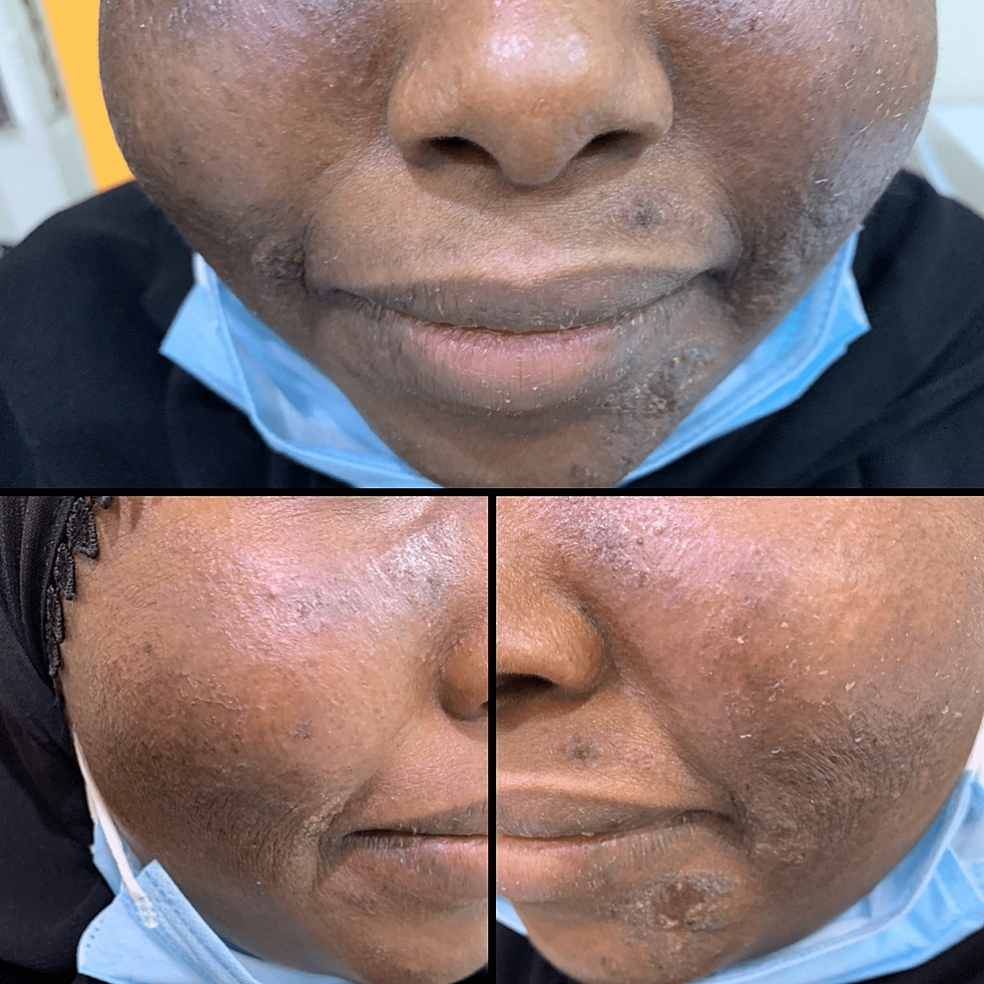
Ten weeks after the start of the treatment. A complete resolution on the cheeks with a minor scaly lesion under the left corner of the mouth.

At 10 weeks, the patient showed complete resolution of the lesions on the cheeks, with only a small scaly plaque still present under the mouth (Figure 4). Finally, a complete resolution was achieved two months after the initiation of the therapy.

## Discussion

This paper has two specific aims. The first aim is to raise awareness of the possible development of a rare side effect related to the use of isotretinoin. Second, to help avoid any delay in the initiation of proper therapy due to a significant side effect that affects the patient not only physically but mentally as well.

In our case, the patient developed itchy, adherent yellow, scaly plaques on her cheeks shortly after the initiation of isotretinoin for her acne. She has never experienced any similar dermatological manifestations before. To our knowledge, only a few cases with more or less a similar presentation have been documented previously in the literature. A retrospective study published in 2012 by Barzilai et al. presented five patients aged between 18 and 23 years who experienced a similar rash. The rash had flat-topped or spiky, tiny, yellow, adherent, greasy scales that were predominantly on the cheeks. It was defined as seborrheic dermatitis that developed during or after the start of the isotretinoin course [5]. As this study in the literature has a very similar dermatological presentation to our case, the report of this study is in line with our report that isotretinoin could lead to a seborrheic dermatitis-like drug eruption. We approached our patient accordingly and the lesions completely resolved in 2 months.

Finally, since our patient did not take any other medication besides isotretinoin, had no previous history of a similar presentation, and did not consume any suspicious food around the time the lesions formed, we were able to determine that the development of this seborrheic dermatitis-like eruption was caused by isotretinoin. Although this is a rare side effect, dermatologists should be aware of this eruption as it significantly affects the patient physically and mentally. Also, they should keep this eruption in mind to avoid misdiagnosis or any delay in the initiation of the right therapy.

## Conclusions

Isotretinoin is widely used in the management of acne vulgaris. It is known to have numerous severe side effects that affect patients physically and mentally, such as seborrheic dermatitis-like eruptions on the face. Dermatologists should be aware of this mentally and physically debilitating side effect to treat patients properly and promptly.
